# Development of Submicrocapsules Based on Co-Assembled Like-Charged Silica Nanoparticles and Detonation Nanodiamonds and Polyelectrolyte Layers

**DOI:** 10.3390/pharmaceutics14030575

**Published:** 2022-03-05

**Authors:** Konstantin V. Palamarchuk, Tatiana N. Borodina, Anastasia V. Kostenko, Yury M. Chesnokov, Roman A. Kamyshinsky, Natalya P. Palamarchuk, Elena B. Yudina, Elena D. Nikolskaya, Nikita G. Yabbarov, Mariia R. Mollaeva, Tatiana V. Bukreeva

**Affiliations:** 1 National Research Centre “Kurchatov Institute”, 1 Akademika Kurchatova Sq., 123182 Moscow, Russia; nas-kostenko@mail.ru (A.V.K.); chessyura@yandex.ru (Y.M.C.); kamyshinsky.roman@gmail.com (R.A.K.); nat_pal1239@mail.ru (N.P.P.); bukreeva@crys.ras.ru (T.V.B.); 2 Shubnikov Institute of Crystallography of Federal Scientific Research Centre “Crystallography and Photonics” of Russian Academy of Sciences, 59 Leninsky Pr., 119333 Moscow, Russia; borodina@crys.ras.ru; 3 Moscow Institute of Physics and Technology, 9 Institutskiy Per., 141701 Dolgoprudny, Russia; 4 Ioffe Institute, 26 Politekhnicheskaya Str., 194021 St. Petersburg, Russia; yudina@mail.ioffe.ru; 5 N.M. Emanuel Institute of Biochemical Physics of Russian Academy of Sciences, 4 Kosygina Str., 119334 Moscow, Russia; elenanikolskaja@gmail.com (E.D.N.); yabbarovng@gmail.com (N.G.Y.); mollaevamariia@gmail.com (M.R.M.)

**Keywords:** colloidosomes, nanoparticles interaction, Pickering emulsions, nanodiamonds, layer-by-layer adsorption, thymoquinone, encapsulation

## Abstract

Capsules with shells based on nanoparticles of different nature co-assembled at the interface of liquid phases of emulsion are promising carriers of lipophilic drugs. To obtain such capsules, theoretically using the Derjaguin–Landau–Verwey–Overbeek (DLVO) theory and experimentally using dynamic light-scattering (DLS) and transmission electron microscopy (TEM) methods, the interaction of like-charged silica nanoparticles and detonation nanodiamonds in an aqueous solution was studied and their ratios selected for the formation of submicron-sized colloidosomes. The resulting colloidosomes were modified with additional layers of nanoparticles and polyelectrolytes, applying LbL technology. As a model anti-cancer drug, thymoquinone was loaded into the developed capsules, demonstrating a significant delay of the release as a result of colloidosome surface modification. Fluorescence flow cytometry and confocal laser scanning microscopy showed efficient internalization of the capsules by MCF7 cancer cells. The obtained results demonstrated a high potential for nanomedicine application in the field of the drug-delivery system development.

## 1. Introduction

One of the promising drug-delivery vehicles is microcapsule colloidosomes based on emulsions stabilized by solid colloidal particles—Pickering emulsions [1,2,3]. The use of nanoparticles of various natures (organic, inorganic) and functionalities (magnetic [4,5], plasmon-resonant [6], fluorescent [7], etc.) allows the design of complex systems for theranostics. The structure and permeability of the nanoporous shells of such colloidosomes can be regulated by nanoparticle size, shape, surface properties [8,9,10], and additional layers (e.g., polyelectrolyte layers [11]), allowing control of the release of encapsulated substances.

Colloidosomes based on oil-in-water emulsions can be used to deliver hydrophobic drugs [12], which cannot be independently transported in the bloodstream. The high stability and low toxicity of capsules based on Pickering emulsions make them preferable for systems derived from surfactant-based emulsions [1]. The high thermodynamic stability of the colloidosomes is provided by a decrease in total free energy since, under the condition of partial wetting by both phases, colloidal particles are self-assembled at the interface between two immiscible liquids, and the fluid–fluid surface area is replaced by a particle–fluid surface area [13,14]. Meanwhile, a layer of solid particles prevents the coalescence of droplets. However, colloidal particles rarely possess dual wettability, and additional surfactants are usually added to the particles [15], making the system undesirable for biomedical application due to surfactant toxicity. An alternative approach is the use of heteroaggregates of nanoparticles, whose surface properties ensure their self-assembly at liquid–liquid interfaces for emulsion stabilization [16]. Generally, heteroaggregates for Pickering emulsion stabilization are formed from oppositely charged nanoparticles that easily assemble due to electrostatic attraction [16,17,18]. Although the co-assembly of the like-charged nanoparticles of different types is also possible as a result of dispersion interactions [19], this approach has never been applied for the colloidosome formation. The development of such systems may expand the possibilities of the production of multifunctional capsules based on Pickering emulsions.

The preparation of colloidosomes with the shell of the nanoparticles of different functionalities can provide versatile applications for the obtained system [20]. Due to specific physicochemical and physical properties of detonation nanodiamonds (DNDs), they are the promising component of the colloidosome shell [21,22,23] for biomedical application. DNDs have high monodispersity, excellent biocompatibility, and chemical inertness, combined with the possibility of controlled surface functionalization. Moreover, DNDs can have photoluminescence via embedded point defects and fluorescence with the possibility of optically detectable magnetic resonance. Colloidosomes with a DND-containing shell could employ all the advantages of these unique nanoobjects for drug delivery, while enclosing a therapeutic agent in the volume of the capsule. Farias et al. developed he colloidosomes from DNDs, but their size was in the micro range, which is not applicable for drug-delivery systems [23]. The work [24] shows the possibility of submicron colloidosome formation from DNDs and stearic acid or stearyl amine. However, in vivo studies have demonstrated that the safety of DNDs is ensured only at very limited doses of their administration [25]. Therefore, the development of submicron colloidosomes with a shell from a mixture of DNDs with less-toxic nanoparticles, for example, silica, that are widely used for biomedical purposes [26], is of interest. Moreover, an additional coating of colloidosomes with biopolymer layers can be used to reduce the toxicity of the obtained system. For this purpose, the layer-by-layer (LbL) method is a simple and convenient procedure to modify the colloidosome surface. The LbL technique allows the creation of a multilayer shell by alternating the adsorption of polyelectrolytes, nanoparticles, etc., mainly due to electrostatic interaction [27]. Moreover, LbL assembly can be applied for the formation of multipurpose capsules, the shell of which may include natural inorganic nanotubes [28], smaller polyelectrolyte containers [29], and even live cells [30]. The surface modification of colloidosomes by different polyelectrolytes can provide biocompatibility properties to the system, as demonstrated on inorganic nanoparticles covered with various polymers [31].

LbL capsules have been actively studied over the past 20 years due to the broad possibilities for adjusting the thickness, permeability, and biodegradability of their shells [32,33]. However, only a few works have described colloidosomes based on Pickering emulsions modified with multilayer polyelectrolyte shells [11,34,35]. It has been shown that additional polymer layers allow the control of the permeability of capsules [11]. Moreover, the polyelectrolyte shell provides an increased stability of colloidosomes, for example, during centrifugation [35]. However, the above-mentioned papers described colloidosomes in the micrometer size range, with the shells formed by monoparticles [11,34,35].

In this work, we studied the formation of submicrocolloidosomes with shells consisting of a mixture of similarly charged DND and Ludox Cl nanoparticles. SiO_2_ nanoparticles coated with Al_2_O_3_ were chosen for co-assembly with DNDs due to their increased tendency to aggregate [19]. The aim of this work was to study the possibility of colloidosome stabilization with a mixture of like-charged nanoparticles, and to perform the LbL deposition of a polyelectrolyte shell on the surface of the obtained colloidosomes to regulate the release of the encapsulated substance and reduce the cytotoxicity of the capsules.

## 2. Materials and Methods

### 2.1. Materials

Ludox Cl (LCl) colloidal silica (30 wt.% suspension in H_2_O) (Sigma–Aldrich, St. Louis, MO, USA); soybean oil (Sigma–Aldrich, St. Louis, MO, USA); chitosan, low molecular weight (Sigma–Aldrich, St. Louis, MO, USA); sodium salt of alginic acid (Sigma–Aldrich, Dorset, UK); hydrochloric acid 99.9% (SigmaTek, Khimki, Russia); sodium hydroxide 99.9% (Sigma–Aldrich, Hamburg, Germany); Thymoquinone (TQ) (2-isopropyl-5-methyl-1,4-benzoquinone, 99%) (Sigma–Aldrich, Hamburg, Germany); Nile Red (Sigma–Aldrich, Hamburg, Germany); phosphate buffered saline (PBS) (Biolot, St. Petersburg, Russia); 3-(4,5-dimethyl-2-thiazolyl)-2,5-diphenyl-2H-tetrasolium bromide (MTT) and Mowiol (Sigma–Aldrich, St. Louis, MO, USA); 96% ethanol (Chimmed, Moscow, Russia); Dulbecco’s modified eagle’s medium (DMEM) (Gibco, Waltham, MA, USA); fetal bovine serum (FBS) (Gibco, Waltham, MA, USA); 0.9% saline (PanEco, Moscow, Russia); dimethyl sulfoxide (DMSO) (Amreso, Solon, OH, USA); 0.02% EDTA and 0.05% trypsin solutions (Gibco, Waltham, MA, USA); gentamycin (PanEco, Moscow, Russia), p-formaldehyde and glycine (Merck, Darmstadt, Germany). All chemicals were used without future purifications. Milli-Q water was used in the experiments.

DND hydrosol was prepared as described in [36] from industrially purified detonation nanodiamond powder purchased from Federal State Unitary Enterprise “Technolog” (St. Petersburg, Russia). Detailed technique is given in SI. Dynamic light-scattering measurements of the DND hydrosol are presented in Figure S1.

### 2.2. Theoretical Study of the Nanoparticles Interaction

The total interaction energy (U) was described using the extended theory of Derjaguin–Landau–Verwey–Overbeek (DLVO) [37]:

(1) $$U(h)=U_{e}(h)+U_{m}(h)+U_{s}(h),$$

where *h*—distance between the particles (nm), *U_e_*(*h*) and *U_m_*(*h*)—energy of electrostatic and intermolecular interaction of the nanoparticles, and, accordingly, *U_s_*(*h*)—structural component of repulsion due to disjoining pressure.

To evaluate the energy of electrostatic interaction between spherical particles of different natures, the following equation was used:

(2) $$U_{e}(h)=2\pi \varepsilon \varepsilon_{0}\zeta_{1}\zeta_{2}r\ln(1+\exp(-\chi h)),$$

where *r* = *r*_1_ + *r*_2_, *r*_1_, *r*_2_—radius of nanoparticles (nm), *ε*—medium permittivity, *ε*_0_—vacuum permittivity (F·m^−1^), *χ*—Debye length (nm^−1^), and *ζ*_1_, *ζ*_2_—zeta potential of the particles (mV).

The calculation of intermolecular interaction was carried out according to the Hamaker equation:

(3) $$U_{m}(h)=-\frac{A_{12}}{6}(\frac{2r_{1}r_{2}}{h^{2}+2r_{1}h+2r_{2}h}+\frac{2r_{1}r_{2}}{h^{2}+2r_{1}h+2r_{2}h+4r_{1}r_{2}}+\ln\left(\frac{h^{2}+2r_{1}h+2r_{2}h}{h^{2}+2r_{1}h+2r_{2}h+4r_{1}r_{2}}\right)),$$

where *A*_12_—Hamaker constant (J).

(4) $$A_{12}=\sqrt{A_{11}A_{22}},$$

where *A*_11_ and *A*_22_—Hamaker constant for identical phases interacting in water.

The structural component was determined according to the following equation:

(5) Us(h)=πrKsl2e−hl,

where *l*—correlation length (nm), and *K_s_*—parameter of the intensity of structural forces (J/m^3^) associated with the ordering and orientation of dipole solvent molecules. *K_s_* was found experimentally through the efficiency factor of collision between particles at the rapid coagulation threshold.

The efficiency factor for particle aggregation is determined through the ratio of the energy of Brownian motion to the maximum potential barrier between the particles:

(6) $$\Omega =\frac{kT}{U(h^{*})},$$

where *k*—Boltzmann constant (J·K^−1^), and *T*—temperature (°K).

If *U*(h*) > *kT* (the potential barrier is insuperable for particles with energy *kT*), then aggregation is slow, and the efficiency factor Ω < 1. If *U*(h*) ≤ *kT*, then the efficiency factor Ω ≥ 1, and fast aggregation occurs. To determine the structural component, Ω = 1.

### 2.3. Capsule Synthesis

Direct emulsions stabilized by nanoparticles were prepared by the addition of 0.1 M NaOH to 0.25 wt.% dispersions of LCl, DNDs, or their mixtures in a phosphate buffer to adjust pH to 8. Then, the mixture was ultrasonically treated (Hielscher UP400S homogenizer) for 30 s at 20% amplitude and shaken on an orbital shaker (1000 rpm) for 30 min. Soybean oil was added to the resulting particle dispersion in a ratio of 1:9 and homogenized ultrasonically at 20% amplitude for 2 min.

To obtain the multilayer capsules, 10% emulsion was added to the dispersion of LCl (0.06 wt.%, pH 4) in the ratio of 1:10. The mixture was shaken for 10 min followed by the addition of sodium alginate (10 mg/mL per dispersed phase). The capsules were separated from the solution by centrifugation (3000× *g*, 5 min) and washed 3 times with HCl (pH 4). Following the same procedure, the next layer of chitosan (2 mg/mL, pH 4) was formed on the surface of the capsules.

To obtain capsules with thymoquinone or Nile Red, they were preliminarily dissolved in soybean oil at concentrations of 10 and 100 mg/mL (in the case of TQ for release kinetics and in vitro experiments, respectively) and 0.5 mg/mL (in the case of Nile Red).

### 2.4. Loading and Release of Thymoquinone

The concentration of TQ was analyzed by spectroscopy (Shimadzu UV-3600). To obtain a calibration line, TQ was dissolved in ethanol and then PBS was added to the solution in a ratio of 1:1 under stirring, followed by evaporation of ethanol at 85 °C.

The release behavior of TQ from the capsules was determined in PBS using a dialysis chamber (Serva, 12–14 kDa). The solution was stirred and, after certain time intervals, withdrawn and analyzed spectroscopically. The spectra of thymoquinone were characterized by the presence of one prominent peak (λ_max_) at 254–257 nm.

### 2.5. Dynamic Light-Scattering (DNS) Measurements

The hydrodynamic size and ζ potential were measured by a Zetasizer Nano ZS particle analyzer (Malvern Instruments). Each ζ-potential value was averaged from three subsequent measurement series each of 15 runs, and size value was from five subsequent measurements, each of 20 runs. To measure the ζ-potential in PBS, the following data were used: refractive index—1.33, viscosity—0.88 cPoise, dielectric constant—79. The ζ-potential was calculated from the electrophoretic mobility using the Smoluchowski relationship.

### 2.6. Cryo-Scanning Electron Microscopy (Cryo-SEM)

The morphology of microcapsules was assessed using Versa 3D (Thermo Fisher Scientific, Waltham, MA, USA) cryo-SEM, equipped with PP3010T (Quorum Technologies, Lewes, UK) preparation system. A total of 3 μL of the sample solution was applied to pure C grid glow discharged with PELCO easiGlow (Ted Pella, Redding, CA, USA). Sample vitrification was performed using the Vitrobot Mark IV (Thermo Fisher Scientific, Waltham, MA, USA) with a 10 °C temperature, 95% humidity, and 2.5 s blotting time. Cryo-SEM images were obtained in secondary electron mode with accelerating voltage 30 kV and current 0.28 nA (10 kV and 93 pA). To reveal additional morphological features, samples underwent a sublimation procedure at −60 °C for 15 min in a cryo-SEM chamber.

### 2.7. Cryo-Transmission Electron Microscopy (Cryo-TEM)

For cryo-TEM study, 3 μL of the sample was applied to a Lacey C-only copper grid glow discharged with PELCO easiGlow (Ted Pella, Redding, CA, USA). The vitrification of the samples was carried out using the Vitrobot Mark IV (Thermo Fisher Scientific, Waltham, MA, USA) at 10 °C and 100% humidity; blotting time varied from 1 to 2.5 s. 

Cryo-TEM data were obtained using the Titan Krios 60-300 TEM/STEM (Thermo Fisher Scientific, Waltham, MA, USA). Cryo-electron tomography (cryo-ET) data were collected in an automated mode using Tomography 4 software (Thermo Fisher Scientific, Waltham, MA, USA) with the following parameters: angular step 2°, range [−60°, 60°], number of images 61, total dose ~75 e/Å^2^. Tilt series were obtained at 3800× (18,000×) magnification with defocus value [−15; −25] µm ([−5; −8] μm). 

Angular series alignment and tomographic reconstruction using simultaneous iterative reconstruction (SIRT) were performed using IMOD software [38].

### 2.8. Cell Culture

Human mammary adenocarcinoma MCF-7 cells and immortalized human fibroblasts BJ-5ta were maintained in plastic flasks in the DMEM medium supplemented with 10% fetal bovine serum and gentamycin (50 µg/mL) at 37 °C in a humidified atmosphere containing 5% CO_2_. The cells were replated with trypsin-EDTA solution twice each week.

### 2.9. Cytotoxic Activity Analysis

The capsule stock solutions were sonicated with low-intensity ultrasound before addition to cells.

To assess the cytotoxic activity, the cells were seeded into 96-well plates (4000 cells per well) 24 h before experiment. The capsules TQ and TQ were added to the cells at concentrations from 0.01–100 µM (TQ) and equivalent amounts of blank capsules in triplets, and then incubated under standard conditions for 72 h. The cell survival was determined using the MTT test [39]. Four hours before the end of the incubation, 50 µL of MTT solution in the culture medium (1 mg/mL) was added to each well. Upon color development, the medium was removed, precipitated formazan crystals were dissolved in 100 µL of DMSO, and the color intensity was determined by absorption at 540 nm with a microplate reader. Cell viability was determined as a percentage of the untreated control. Survival curve plotting, IC_50_ value calculation, and statistical analysis were performed in Excel (Microsoft Corporation, Redmond, Washington, DC, USA) and OriginPro (version 2020b, OriginLab Corp., Northampton, MA, USA).

### 2.10. Flow Cytometry Analysis

10 × 10^4^ MCF-7 cells were plated in a 35 mm Petri dish in 1.75 mL of media 24 h before the experiment. A total of 250 µL of capsules with Nile Red (1% vol., 0.05 mg/mL dye) were added to the cells for 2 h. After incubation, the cells were detached with trypsin-EDTA, rinsed twice with cold PBS, and collected by centrifugation at 500× *g*. The fluorescence intensity of the cells was measured with Dako CyAn ADP flow cytometer equipped with a 635 nm laser and a 665 nm band-pass filter. For each sample (10^5^ cells), the mean fluorescence intensity (MFI) was determined.

### 2.11. Confocal Microscopy Analysis

The MCF-7 cells were plated onto cover glasses into 24-well plates (2 × 10^4^ cells in 1 mL of culture medium per well) the day before the experiment. Then, 125 µL of capsules with Nile Red (1% vol., 0.05 mg/mL dye) were added to cells for 2 h. After incubation, the cells were washed thrice with PBS, fixed in 2% p-formaldehyde, washed twice with PBS supplemented with 50 mM glycine to decrease the autofluorescence, and embedded in Mowiol. The fluorescence intensity and distribution of capsules in the cells were studied using a Carl Zeiss Cell Observer Z1 confocal microscope with a 100× objective. The photos were finally processed with Bitplane Imaris 7.2.3 (Bitplane AG, Zürich, Switzerland) and Adobe Photoshop CS3 (Adobe Systems Inc., Mountain View, CA, USA).

## 3. Results and Discussion

**Figure 1:**
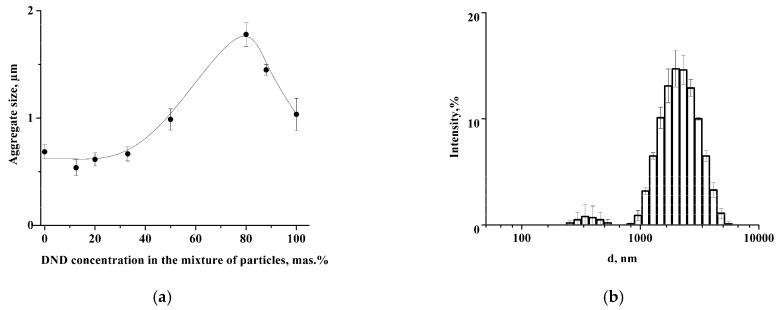
(**a**) Dependence of the hydrodynamic size of the aggregates on the DND content (PBS, pH 8); (**b**) the size distribution of the aggregates at 80 wt.% of DNDs in particle mixture.

We studied the possibility of colloidosome stabilization using a mixture of LCl and DND nanoparticles in an aqueous medium, where they are similarly charged. It was shown that LCl and DNDs possess the same negative charge in PBS at pH 8: ζ_LCl_ = −22.0 ± 2.2 mV, ζ_DND_ = −22.2 ± 0.4 mV. DLS data demonstrate the formation of the aggregates with an average size of 660 nm in the mixture of nanoparticles with DND content ≤ 33 wt.% (Figure 1a). The increase of DND concentration to 80 wt.% leads to growth of the average size of aggregates to 1.8 ± 0.1 μm. At the same time, the DLS histogram also contains a small peak at 350 ± 18 nm (Figure 1b). The further increase of the DND content results in a reduction of aggregate size to 1 μm (Figure 1a). The values of the ζ-potential of the aggregates were around −22 mV regardless of the nanoparticle mass ratio (Figure S2), which indicates charge stability of the nanoparticles in the aggregates.

**Figure 2:**
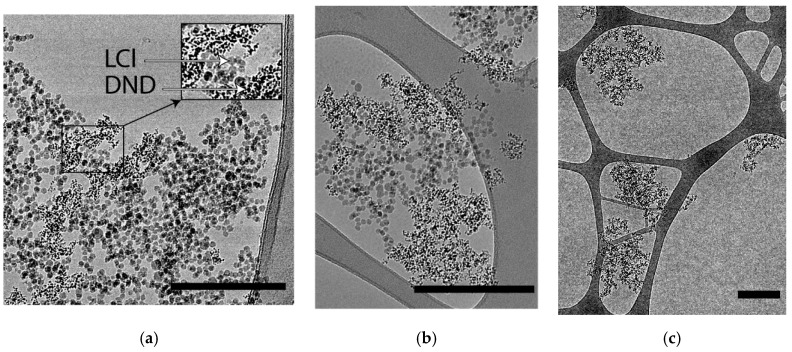
Cryo-TEM images of the aggregates in LCl/DND mixture with DND content 12.5 wt.% (**a**) and 80 wt.% (**b**,**c**). The arrows (**a**) indicate LCl and DND particles. Scale bars are 400 nm (**a**,**b**) and 1 µm (**c**).

Cryo-TEM images demonstrate the formation of nanoparticle clusters in LCl/DND mixtures (Figure 2). Figure 2a shows compact clusters of nanodiamonds in the sample with 12.5% of DNDs. Increase of DND content to 80% results in the presence of isolated DND aggregates, which corresponds to the peak at 350 nm in DLS histogram (Figure 1b).

**Figure 3:**
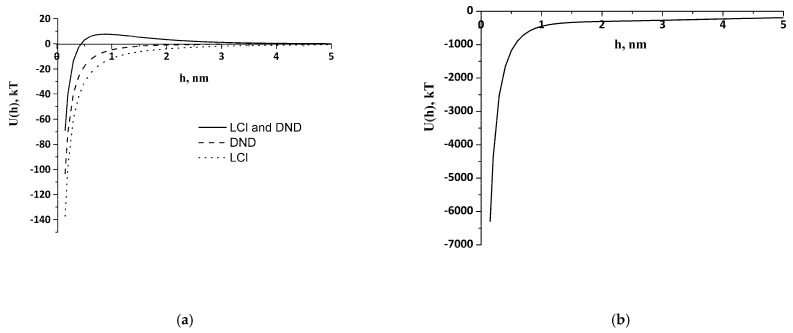
Energy of interaction: (**a**) between the nanoparticles; (**b**) between the clusters of the LCl and DND nanoparticles.

Theoretical study of the interaction of like-charged LCl and DND particles was carried out, applying the extended DLVO theory. Potential curves were plotted using experimental data obtained by the DLS and cryo-TEM, as well as literature data for the Hamaker constant from [40] (Table 1). The electrostatic factor was calculated using Formula (2) and the dispersion interaction through the Hamaker Equation (3). The structural component was evaluated by Formula (5), where l was taken as 1 nm, and the intensity parameter *K_s_* was found through the particle-collision efficiency factor Ω. We considered the aggregate–particle interaction and applied Formula (6) under the condition Ω = 1, which corresponds to the threshold of rapid coagulation during aggregate formation. To calculate *K_s_*, *U*(*h*) was equated to *kT*, the aggregate size was taken as *r_1_*, and the individual particle size was taken as *r_2_*. Nanoparticles of the same nature were found to have barrier-free interaction and an absence of structural factors under experimental conditions (Figure 3a), which leads to the formation of clusters.

Clusters of 350 nm of DNDs, 660 nm of LCl, and their 1.8 μm aggregates were taken into account to calculate the DND/LCl interaction. Parameters *K_s_* and *A*_12_ for the interaction of clusters were the same for the interaction of individual particles (Table 1). Figure 3a demonstrates a potential barrier of 7.6 *kT* on the LCl–DND interaction curves, which can explain the lack of heteroaggregate formation between individual LCl and DND nanoparticles in PBS at pH 8. It can be seen that only a certain size of cluster makes the aggregation an energetically favorable process (Figure 3b).

**Figure 4:**
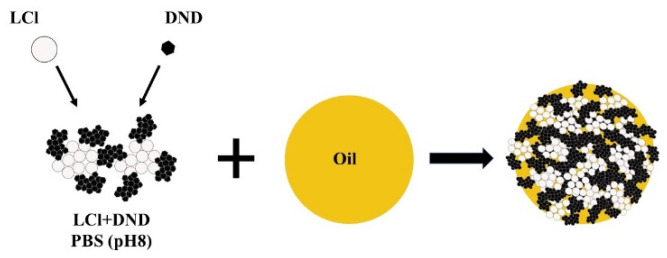
Scheme of stabilization of the direct emulsion by the LCl/DND mixture.

Based on the above results, it can be assumed that the stabilization of Pickering emulsion with the LCl/DNA mixture will be carried out according to the scheme shown in Figure 4. The same colloidosome shell structure was observed for “dodecane-in-water” emulsion stabilized by the mixture of magnetite nanoparticles and fluorescent silica [20].

**Figure 5:**
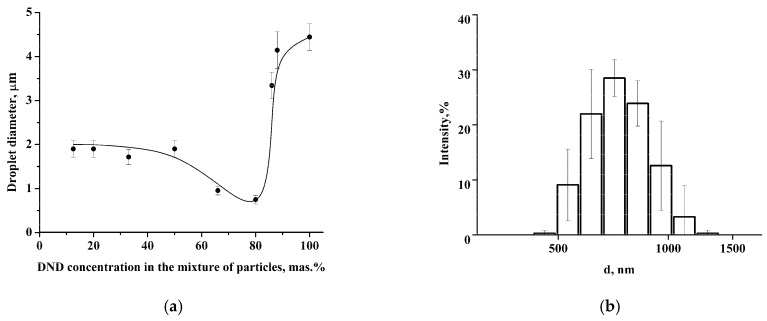
(**a**) Dependence of the Pickering emulsion droplet size on the DND content in the mixture of the nanoparticles; (**b**) size distribution of the Pickering emulsion droplets at 80 wt.% of DNDs in the mixture of nanoparticles.

Coalescence-resistant Pickering emulsion was obtained by stabilizing soybean oil droplets with a mixture of LCl and DND nanoparticles. The size of colloidosomes (droplets of the oil phase with the nanoparticle shell) depends on the DND content in the mixture of nanoparticles (Figure 5a). It was found that the colloidosome diameter at a DND concentration of 10–50 wt.% is about 2 μm. The further increase of DND concentration up to 80 wt.% leads to a decrease of the average diameter of colloidosomes to 750 nm (Figure 5b) followed by growth to 4–4.5 μm with a subsequent increase of DND content in the mixture.

**Figure 6:**
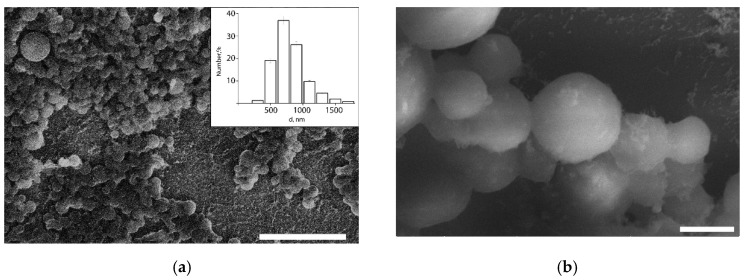
Cryo-SEM images (**a**,**b**) and size distribution of the colloidosomes (inset in (a)) with the shell of the nanoparticle mixture with 80 wt.% of DNDs. Scale bars are 10 μm (**a**) and 1 µm (**b**).

Thus, colloidosomes with minimum size were formed using the mixture of like-charged LCl and DND nanoparticles with the 80 wt.% DNDs concentration, in which an increased tendency of nanoparticles towards aggregation was observed, i.e., under these conditions, the interaction of the nanoparticle clusters is so strong that its provides stabilization to soybean oil droplets in the submicron size range. DLS data were confirmed by the cryo-SEM study (Figure 6). The obtained colloidosomes have typical spherical shape. It was shown that in a nanoparticle mixture with 80 wt.% of DNDs, approximately 83 numb.% of the colloidosomes are smaller than 1 µm in diameter, and their average diameter is 820 nm. Only a few large colloidosomes with size up to 3 µm were observed.

Submicron colloidosomes present wider possibilities for their use in biomedical application compared to the microsized capsules. The submicron size increases the efficiency of capsule penetration into small capillaries in the bloodstream and increases the efficiency of internalization into cells. However, Pickering emulsion-based colloidosomes are known to have a porous shell structure, which increases the release rate of the encapsulated substance and reduces the efficiency of encapsulation [3,8,9,10,11]. In this regard, colloidosome shells were modified with additional layers of the nanoparticles and polyelectrolytes using the layer-by-layer method, and the effect of this modification on the capsule size, as well as on the release rate of the model drug, was studied.

**Figure 7:**
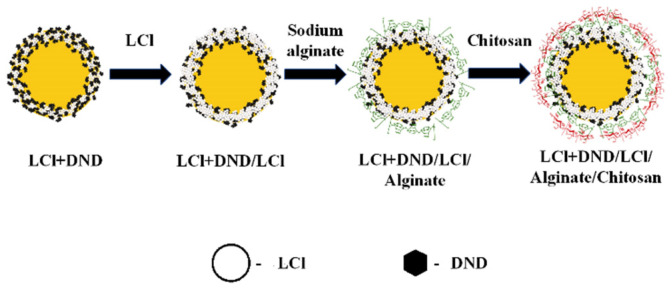
The scheme of the capsule formation.

Figure 7 shows the scheme of the capsule formation. To create a multilayer shell, LCl nanoparticles, positively charged at pH 4, were applied as the first additional layer on the colloidosomes. The use of nanoparticles, in contrast to polyelectrolytes, did not lead to significant aggregation of emulsion droplets. In addition, it was shown in [11] that additional adsorption of nanoparticles on the shell of the colloidosomes based on Pickering emulsion leads to a rearrangement of the shell, which contributes to the closure of pores and an increase in the efficiency of encapsulation.

**Figure 8:**
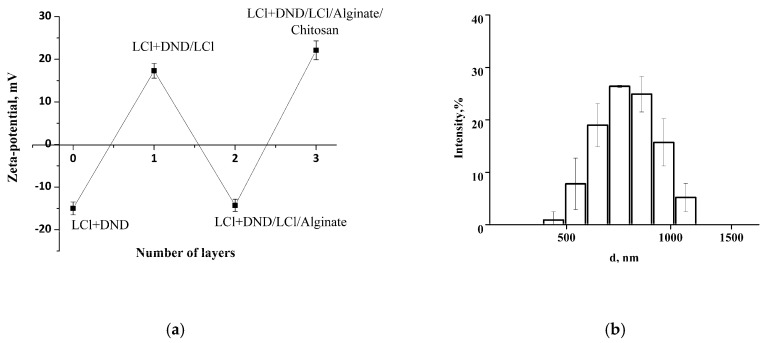
(**a**) The zeta-potential change as a result of multilayer shell formation (pH 4); (**b**) the size distribution of the (LCl + DND)/LCl/alginate/chitosan capsules.

Initial colloidosomes with the shell of co-assembled LCl and DNDs at pH 4 possess negative zeta potential of −16 mV, which presents the possibility to electrostatically adsorb LCl nanoparticles positively charged at pH 4. The zeta potential of the resulting colloidosomes with additional LCl layer was revised to +17 mV. Then, alginate and chitosan were successfully deposited on the surfaces of the colloidosomes, which was confirmed by the recharge of the capsule surface (Figure 8a). The hydrodynamic size of the capsules was not significantly changed after polyelectrolyte adsorption (Figure 8b compared to Figure 5b), which confirms the porosity of initial shell and indicates the adsorption of LCl nanoparticles in shell pores, as well as the formation of a thin polyelectrolyte layer without capsule aggregation.

**Figure 9:**
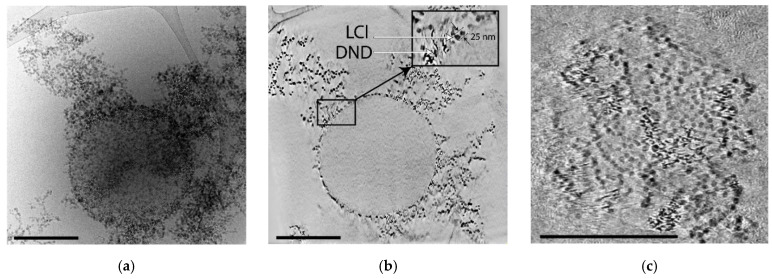
Cryo-TEM image (**a**) and tomographic slices (**b**,**c**) of (LCl + DND)/LCl/alginate/chitosan microcapsule; (**b**) slice through the center of the microcapsule; (**c**) slice through the upper boundary. Scale bar is 400 nm.

Figure 9 demonstrates cryo-TEM images of the capsule, where it can be clearly seen that the nanoparticles form a solid clustered shell. The tomographic slices demonstrate the presence of gaps in the shell with size comparable to 2–3 LCl nanoparticles (Figure 9b,c) and larger voids, which can be associated with the desorption of the nanoparticles during the sample preparation, since ordered columns of nanoparticles are adjacent to the capsule. The tomographic section through the center of the capsule (Figure 9b) demonstrates that a ~30 nm thick shell, consisting of a monolayer of LCl and clusters of DND nanoparticles, is formed on the surface of the oil core, with nanoparticles weakly immersed in oil. Extended clusters of nanoparticles, which may have been formed from the shells of other capsules destroyed due to the peculiarities of the vitrification procedure, can also be observed around the capsule.

**Figure 10:**
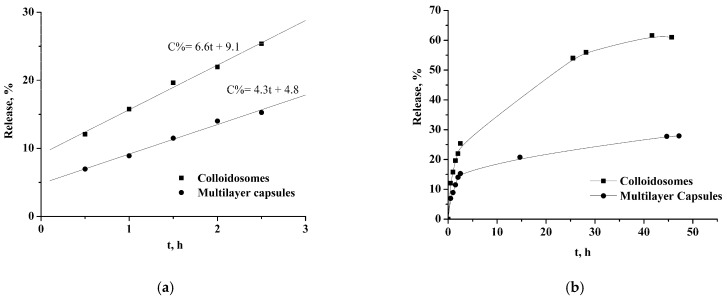
The release of TQ from colloidosomes and multilayer capsules at 2.5 h (**a**) and 45 h (**b**).

TQ was encapsulated into the obtained system as a model hydrophobic drug to study the effect of colloidosome modification by nanoparticles and polyelectrolyte layers on the release rate of the encapsulated substance. TQ encapsulation efficacy was 90 ± 5%, and its inclusion had no effect on the size of the capsules (Figure S2). When studying the kinetics of TQ release from the initial colloidosomes and the multilayer capsules based on them, it was found that the release of TQ occurs according to the 0th-order equation in the first 2.5 h (Figure 10a). The ratio of the release rate constants for monolayer and multilayer shells is approximately equal to 1.5. The initial release profile did not start from zero as a result of a partial release of the substance from the capsules before measurements, due to the permeability of the shell. The release of TQ from colloidosomes characterized by the “initial-burst effect” is followed by a slowing down, when 61% and 28% of TQ were found in the supernatant in 45 h for the initial colloidosomes and multilayer capsules, respectively. Thus, the formation of the additional shell of nanoparticles and polyelectrolytes significantly slowed the release of the drug.

**Figure 11:**
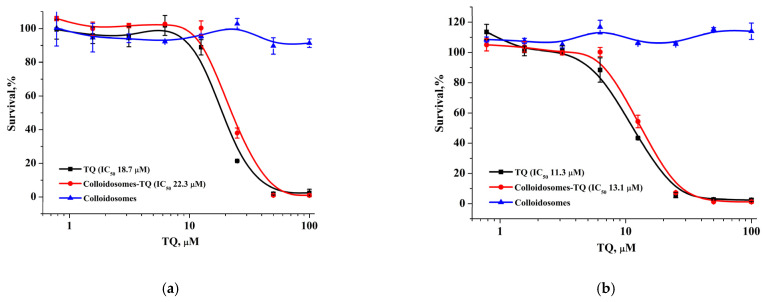

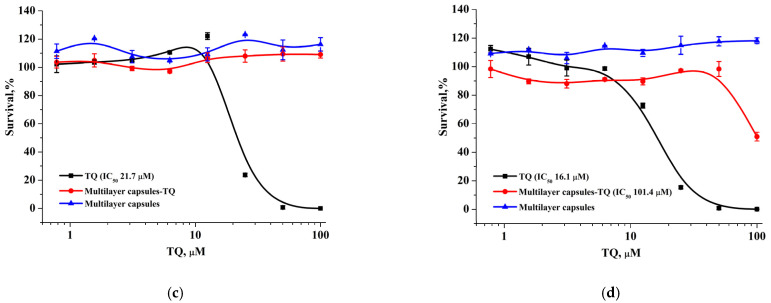
MCF-7 (**a**,**c**) and BJ-5ta (**b**,**d**) cell survival after 72 h incubation with the TQ colloidosomes, TQ-loaded capsules, and blank capsules.

The modification of the initial colloidosomes with LCl nanoparticles and the biocompatible polyelectrolytes can reduce the toxicity of the final capsules loaded with the drug. For verification, we examined the cytotoxic activity of hydrophobic thymoquinone (TQ), TQ-loaded capsules, and empty capsules using the MTT test against MCF-7 and BJ-5ta cells (Figure 11). TQ-loaded microcapsules displayed an overall similar activity against both cell lines (IC_50_ values: 18.7–21.7 µM for MCF-7 and 11.3–16.1 µM for BJ-5ta) and the same level of cytotoxicity as the TQ solution in the DMSO, which may be explained by a weak TQ retention in unmodified capsules and a rapid TQ release (Figure 11a,b). Furthermore, we found that capsules with a modified surface had the opposite effect on cytotoxicity: the encapsulation of TQ in modified capsules significantly reduced its antitumor activity in both cell lines. BJ-5ta cells were more sensitive to TQ-loaded modified capsules with relatively low IC_50_ (101.4 µM), which was 10-fold lower than for TQ solution in DMSO, while MCF-7 cells showed an approximately 100% survival rate at the studied concentration range (Figure 11c,d). At the same time, both unmodified and modified empty capsules did not reveal any cytotoxic activity.

The results obtained indicate a significant effect of the additional polyelectrolyte layers on capsules on TQ release and cytotoxic activity of encapsulated TQ, and confirms the data concerning drug release in vitro.

**Figure 12:**
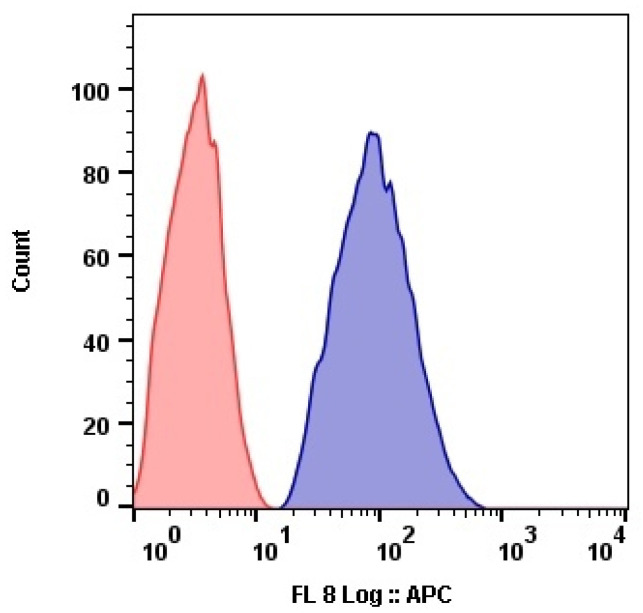
MCF7 cells MFI after 2 h incubation with the Nile Red-labeled capsules. Untreated cells (red).

The submicron size of the prepared capsules facilitated their efficient internalization by cells. The results of flow cytometry analysis showed that MCF-7 cells internalized Nile Red-labeled capsules at high concentration (Figure 12).

**Figure 13:**
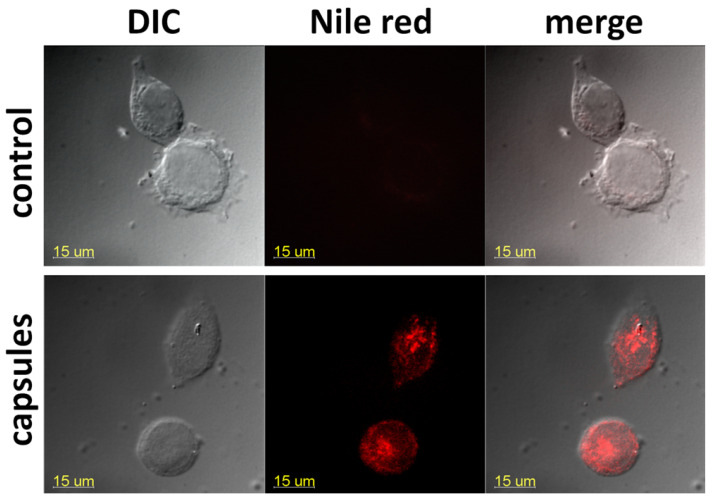
MCF7 cell fluorescence after 2 h incubation with the Nile Red-labeled capsules (bottom row). Untreated control—upper row. Bar—15 µm.

Flow cytometry results agreed with confocal microscopy data: we observed a compartmentalized fluorescence of the dye-labeled capsules in the perinuclear cell area, demonstrating an effective microcapsule internalization (Figure 13).

## 4. Conclusions

Colloidosomes based on nanodiamonds suggest promising drug-delivery systems, which must have submicron size, nontoxicity, and controlled shell permeability. We propose the strategy for submicrocapsule fabrication using co-assembly of nanodiamonds and silica at the interface between the emulsion liquid phases and LbL deposition of the additional layers of nanoparticles and polyelectrolytes onto Pickering emulsion droplets to control the properties of the capsule shell. Study of the co-assembly of silica nanoparticles and DNDs by the DLS and TEM methods and theoretical evaluations of the interaction energy in aqueous medium under conditions of the same negative electrokinetic potential of the particles shows the formation of clusters of monoparticles and energetically favorable aggregation in the system upon reaching a certain size of such clusters. The clusters stabilize soybean oil droplets in water with the formation of the submicrocolloidosomes under such conditions at a LCl:DND mass ratio of the nanoparticles of 1:4. Additional layers of silica, chitosan, and alginate were successfully deposited on the surface of the colloidosomes at pH 4, which was confirmed by the switching of the sign of the electrokinetic potential of particles at each stage of adsorption. The resulting capsules retained their original size in the submicron range, which is a significant achievement for emulsion-based capsules. The LbL surface modification of the colloidosomes slows the release of the encapsulated lipophilic thymoquinone by more than two times. Cytotoxic activity results, combined with flow cytometry and confocal microscopy data, demonstrate that prepared capsules exhibit high potential safety and could be promising vehicles for the delivery of various substances.

## Figures and Tables

**Table 1:** The parameters for the particle pair interaction energy evaluation.

Object	*r*_1,_ nm	*r*_2_, nm	*R_agr_*, nm	*ζi*, mV	*K_s_*, MJ/m^3^	*l*, nm	*A*_12_ × 10^−19^, J
DND	2.5	2.5	175	−22	0	1	4.38
LCl	8	8	330		0		1.50
DND-LCl	2.5	8	900		4.48		2.56
DND-LCl (clusters)	175	330	900		4.48		2.56

## Supplementary Materials

The following supporting information can be downloaded at: https://www.mdpi.com/article/10.3390/pharmaceutics14030575/s1, SI: DND hydrosol preparation technique, Figure S1: Size distribution of DND particles in 0.8 wt.% hydrosol, Figure S2: ζ-potential changing depending on the DNDs content in the mixture of the nanoparticles, Figure S3: The size distribution of the TQ-loaded (LCl + DND)/LCl/alginate/chitosan capsules.
