# Supplementary material for: Development of Submicrocapsules Based on Co-Assembled Like-Charged Silica Nanoparticles and Detonation Nanodiamonds and Polyelectrolyte Layers
Source: Pharmaceutics. 2022 Mar 5;14(3):575. doi: 10.3390/pharmaceutics14030575 (PMC8951451; doi:10.3390/pharmaceutics14030575)
Supplement: Supplementary file 1 [file pharmaceutics-14-00575-s001.zip › pharmaceutics-1614835-SI.pdf]

pharmaceutics

Supplementary Materials: Development of Submicrocapsules Based on Co-Assembled Like-Charged Silica Nanoparticles and Detonation Nanodiamonds and Polyelectrolyte Layers

Konstantin V. Palamarchuk, Tatiana N. Borodina, Anastasia V. Kostenko, Yury M. Chesnokov, Roman A. Kamyshinsky, Natalya P. Palamarchuk, Elena B. Yudina, Elena D. Nikolskaya, Nikita G. Yabbarov, Mariia R. Mollaeva and Tatiana V. Bukreeva

SI. DND Hydrosol Preparation Technique

Additional purification of industrially purified detonation nanodiamond powder from remaining metal and inert impurities was carried out using oxidation in strong acids and subsequent etching in HF and KOH. The reagents residual was washed out by multiple centrifugations. Dried DND powder was annealed in air atmosphere at 450 °C for 3 h. Obtained sample was dispersed in deionized water with an ultrasonic horn under acoustic power of 300 W. Primary DND particles with size of around 4.5 nm were separated by centrifugation at 12,000 rpm for 40 min.

The DND hydrosol was evaporated in rotary evaporator thus obtaining DND powder. Elemental composition studied by energy dispersive X-ray analysis: C – 91.2 wt.%, O – 8.7 wt.%, Al, Cl – 0.1 wt.%.

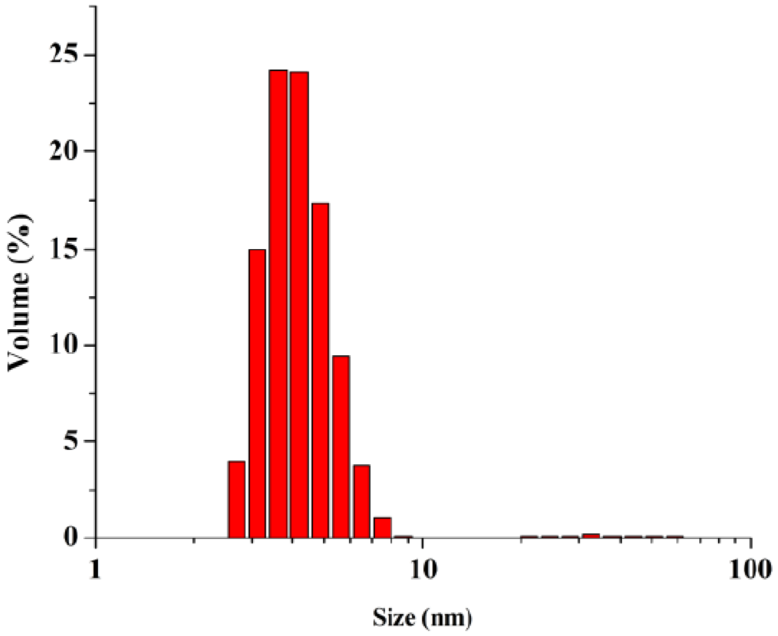

Figure S1. Size distribution of DND particles in 0.8 wt.% hydrosol.

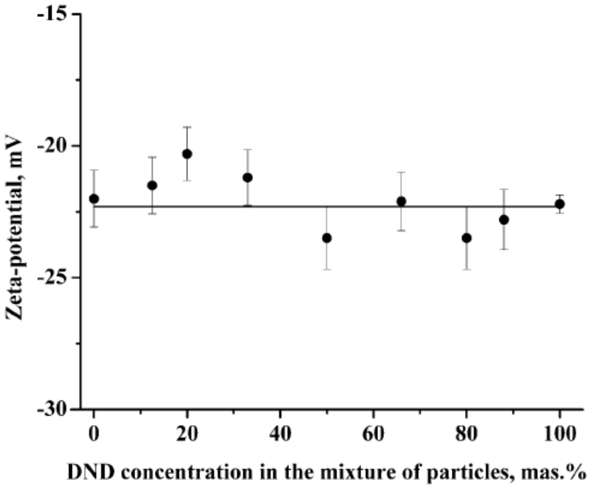

Figure S2. ζ- potential changing depending on the DNDs content in the mixture of the nanoparticles.

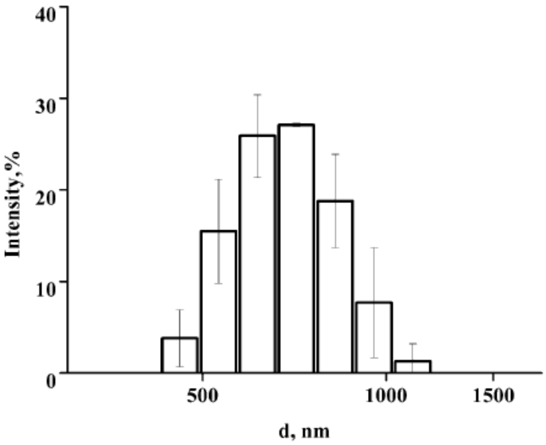

Figure S3. The size distribution of theTQ-loaded (LCl+DND)/LCl/Alginate/Chitosan capsules.
